# Functional Outcome of Platelet-Rich Plasma (PRP) Intra-lesional Injection for Tennis Elbow – A Prospective Cohort Study

**DOI:** 10.7759/cureus.22974

**Published:** 2022-03-08

**Authors:** Madhavan Paramanantham, Hariprasad Seenappa, Sagar Venkataraman, Arun H Shanthappa

**Affiliations:** 1 Orthopaedics, Sri Devaraj Urs Medical College, Sri Devaraj Urs Academy Of Higher Education and Research, Kolar, IND; 2 Orthopaedics, Sri Devaraj Urs Medical College, Sri Devaraj Urs Academy of Higher Education and Research, Kolar, IND

**Keywords:** mayo score, vas, lateral epicondylitis, tennis elbow, prp injection

## Abstract

Introduction

Platelet-Rich Plasma (PRP) is an autologous human plasma preparation with a higher platelet concentration. Injection of PRP were, found to be effective in treating tendinopathy and arthritis. A few studies only focused in using PRP injection in patients with tennis elbow. This study was conducted to access the functional outcome of PRP injection in tennis elbow patients.

Methodology

A prospective study was done from June 2020 to June 2021, at R. L. Jalappa Hospital, Kolar, India among 80 individuals diagnosed with tennis elbow. All individuals aged between 18 to 65 years of either gender and the pain symptoms not subsided with oral analgesics or physiotherapy were included in this study. We analysed all the patients with a MAYO elbow performance score and Visual Analogue Pain Scale (VAS) during the follow-up period.

Results

In total, 80 individuals participated in our study, of which the mean age of the participants was 45.54. There is statistical significance in the difference of means of pain score obtained using both VAS and MAYO score at 12 weeks and 24 weeks. There is high significant positive correlation of age with the pain scores at 12th week and 24th week after the procedure.

Conclusion

In tennis elbow patients, PRP injection shows an effective reduction in pain according to VAS and MAYO score and especially, younger age patients have shown more benefit in terms of pain reduction with PRP treatment.

## Introduction

Platelet-rich plasma (PRP) is an autologous human plasma preparation with a higher platelet concentration obtained by centrifuging a larger volume of the patient's blood. Platelets alpha granules contain a variety of growth factors and mediators [transforming growth factor-1 (TGF-1), platelet-derived growth factor (PDGF), vascular endothelial growth factor (VEGF), epidermal growth factor (EGF), insulin-like growth factor-1 (IGF-1)], that are concentrated during centrifugation to deliver supraphysiologic levels of such growth factors and cytokines to an injury site and aid in the natural healing process [1]. The typical human platelet count is between 150000 to 450000 microL. Concentrated platelets of up to 1000,000 microL, indicating a three to five-fold increase in platelet count, have been shown promising in bone and soft tissue repair [2,3]. Several studies were done using PRP to treat tendon injuries or tendinopathies [4-7]. Many of the cytokines identified in PRP are involved in the signaling pathways that occur during the stages of inflammation, cellular proliferation, and subsequent tissue remodeling that occur during the healing process. PRP may also encourage neovascularization, which increases the blood supply, nutrients required for cells to rebuild injured tissue, as well as bring new cells and eliminate debris. These methods of action could be especially important in chronic tendinopathies when the biological conditions aren't conducive to tissue recovery. PRP injections were found to be effective for treating symptomatic tendinopathy in a recent comprehensive review and meta-analysis [8]. Tennis elbow was initially documented by Runge in 1873, and Henry Morris, writing in the Lancet in 1882, coined the term "Lawn Tennis Arm" [9]. Tendinosis, lateral epicondylitis, and Angiofibroblastic hyperplasia are other names for this condition. It tends to occur in regular tennis players where there is a definite relationship between the late backhand play and forceful wrist extension, as the most popular phrase used. The procedure necessitates the extraction of patient blood, centrifugation, and injection of plasma into the lateral epicondyle at the maximum point of tenderness. It has been documented the results have been positive [10]. In 2011, Thanasas conducted research that found differences between PRP and whole blood injections when it comes to reduction in pain better with PRP injection [11]. Furthermore, major variances between commercially available systems and variations in the technique make it impossible to draw definitive findings concerning the usage of PRP. Research on PRP management in lateral epicondylitis is now encouraging, but additional research is needed to thoroughly verify PRP's usefulness. The aim of the study is to evaluate the functional outcome of PRP injection in tennis elbow patients.

## Materials and methods

A prospective study was done from the period of June 2020 to June 2021, at R. L. Jalappa Hospital, Tamaka, Kolar among 80 individuals diagnosed with tennis elbow (chronic lateral epicondylar tendinopathy). All individuals aged between 18 to 65 years of all gender, and the pain symptoms not subsisted with oral analgesics or physiotherapy were included in the study. Patients undergone elbow surgery, diagnosed with septic arthritis, having a history of alcohol and smoking habits, mentally challenged persons, were excluded from the study. All eligible individuals have been included with informed consent. Ethical clearance was taken from the ethical committee at Sri Devaraj Urs Medical College, Tamaka, Kolar. The PRP was prepared from venous whole blood at the point of care and contained concentrated platelets three-folds. All patients underwent intra-lesional PRP injection over the lateral epicondyle at the point of maximum tenderness. We analyzed all the patients with a VAS and MAYO score during the follow-up period. The data were entered in Microsoft Excel, and results were analyzed in Statistical Package for social sciences (SPSS) IBM Corp. Released 2012. IBM SPSS Statistics for Windows, Version 21.0. Armonk, NY: IBM Corp. The continuous variables were described as mean and standard deviations. The qualitative data were described in terms of frequency and percentage. Paired t-test was done to analyze the association between, the mean pain scale score in (VAS and MAYO) on pre and post-treatment procedures. The correlation was used to assess the relationship between continuous variables.

## Results

**Figure 1:**
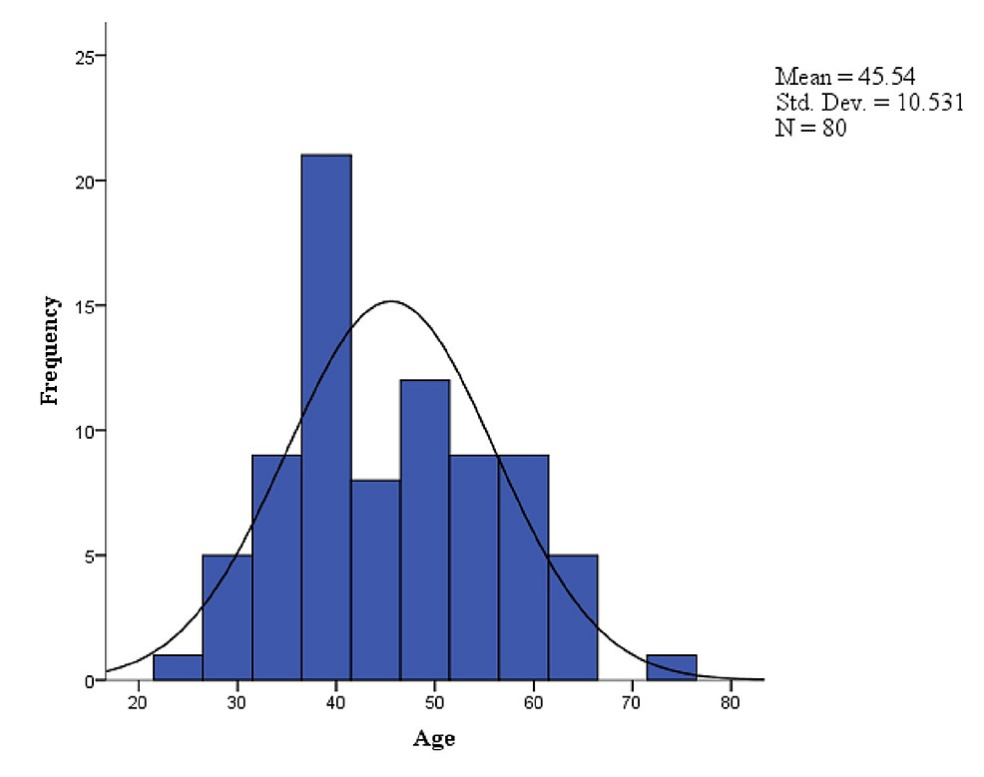
Age distribution of the study participants (n=80)

In total, 80 individuals participated in the study. The mean age of the participants was 45.54, with a standard deviation of 10.531. The baseline characters of the study participants were shown in Figure 1 and Table 1. The mean score value of pain score at various follow-up points is shown in Table 2. By using paired T-test, there is statistical significance in the difference of means of pain score obtained using both VAS and MAYO score (P < 0.001) at 12 weeks and 24 weeks. This association is described in Table 3. There is no significant correlation with MAYO pain scores at the time of pre-procedure but there is a highly significant positive correlation with the pain scores at 12th week and 24th week after the procedure, which signifies the role of age in treatment success. Thus, in patients with tennis elbow, young age has responded well with PRP injection management when compared to increasing age in respect to pain, this correlation is shown in Table 4.

**Table 1:** Baseline characters of the study participants (n=80)

S. No	Baseline characters		Frequency	Percentage
1	Gender	Male	51	63.8
		Female	29	36.3
2	Side of involvement of tennis elbow	Right	66	82.5
		Left	14	17.5

**Table 2:** Distribution of the study participants according to the VAS and MAYO score (n=80) VAS: Visual Analogue Scale

VAS and MAYO score	MAYO pre-procedure	MAYO week 12	MAYO week 24	VAS pre-procedure	VAS week 12	VAS week 24
Mean	63.94	80.63	78.63	6.75	1.60	1.71
Std. Deviation	7.365	6.815	8.073	1.119	0.894	0.957
Median	65	80	80	7	1	2
Inter-quartile range	60 – 70	75 – 85	70 – 85	6 – 8	1 – 2	1 - 2
Mode	70	80	70	6	1	2
Minimum	50	65	60	5	0	0
Maximum	75	95	95	9	4	4

**Table 3:** The associations between the VAS and MAYO pain score before & after the procedure (n=80) VAS: Visual Analogue Scale

VAS and MAYO score before and after intervention		Mean	Correlation significance	T - Value	P - value
Pair 1	MAYO pre-procedure	63.94	.906	-14.976	< 0.001
	MAYO week 12	80.63			
Pair 2	MAYO pre-procedure	63.94	.532	-12.470	< 0.001
	MAYO week 24	78.63			
Pair 3	VAS pre-procedure	6.75	.219	34.577	< 0.001
	VAS week 12	1.60			
Pair 4	VAS pre-procedure	6.75	.262	28.835	< 0.001
	VAS week 24	1.71			

**Table 4:** The correlation between age & MAYO scores at various follow-up periods (n=80)

Variable		MAYO pre-procedure	MAYO week 12	MAYO week 24
Age	Pearson Correlation	- 0.030	0.259^*^	0.348^**^
	P - Value	0.791	0.020	0.002
	N	80	80	80

## Discussion

There is no universal agreement on better PRP preparation in terms of quantity of blood components as currently a variety of commercial PRP devices are available in practice. Prior to centrifugation, whole blood is taken and mixed with an anticoagulant agent, which separates red blood cells (RBCs) from platelet-poor plasma (PPP) and the "buffy coat," which contains high concentrated platelets and leukocytes as well. Platelets are been extracted in a variety of ways and then injected into the lesion or "activated" by adding calcium chloride or thrombin, which causes the platelets to degranulate and release growth factors. The particular makeup of PRP is influenced by both patient-specific factors, such as medications used, and commercial system preparation procedures, and diversity in its composition of PRP preparations causes difficulty in understanding the evidence about the therapeutic efficacy of PRP [2-3,12-13]. PRP has also been hypothesized to promote a regenerative response by inducing a temporary inflammatory event [14]. PRP may have positive immunomodulatory effects on tenocytes, according to other studies [15]. Several meta-analyses on the use of PRP for tendon or ligament injuries have been reported [16-18]. The therapeutic efficacy of PRP against placebo (saline), autologous whole blood, dry needling, and corticosteroids for ligament and tendon injuries are ambiguous and contentious, according to these findings. In contrast to this discussion, this study showed a statistically significant association in the pain scores measured by VAS and MAYO scale on before & after injection of PRP at 12th week and 24th week. Between 8 and 52 weeks, Autologous Blood (AB) and PRP showed a much better improvement than placebo, according to a study done by Krogh et al. [19]. A study done by Watts et al., in the year 2020, compared the efficacy of PRP and surgical outcome in refractory tennis elbow patients, concluded that about 70 percent of the study participants avoided the need for surgery, though post-surgery patients have reduced pain scores than PRP group [20]. A study was done by Yerlikaya et al., in 2017, among 90 patients with lateral epicondylitis, with the aim of comparing the effects of leukocyte-rich and leukocyte-poor PRP on pain and functionality, concluded that there were no significant differences in VAS, patient-rated tennis elbow evaluation, grip and pinch measures, extensor tendon thickness, or cortical derangement across groups (p > 0.05) [21]. A Meta-analysis conducted by Li et al., in China, with seven randomized control trials in 2019 with the aim of comparing the effects of corticosteroids and PRP for treating elbow epicondylitis, conclude that in short term duration corticosteroids show a better result than PRP injection but in long term management, PRP shows better results than corticosteroids therapy in respect to pain reduction and functionality [22]. A Meta-analysis conducted by Sirico et al., in Italy, with four randomized control trials in 2017 with the aim of comparing the effects of autologous blood injections and PRP for treating lateral epicondylitis, conclude that the corticosteroids appear to decrease VAS score in short-term follow-up studies, though the results are not statistically significant. In the mid to long term, no differences were seen. Despite pathophysiological indicators, the presently available data do not support the usefulness of autologous blood injections in medium and long-term follow-up, contrary to the common view among medical experts. More research is needed to determine which treatment has the most influence on pain in lateral epicondylitis [23]. A comparative study was done by Boden et al., in 2019, in the USA among 62 patients retrospectively, with the aim of comparing PRP injection with Tenex for the management of golfers and tennis elbow found that the visual analog pain scale levels, Quick Disabilities of the Arm, Shoulder, and Hand scores, and EuroQol-5D scores all improved clinically and statistically in the PRP and Tenex groups. There was no statistically significant difference between the two groups [24]. Few review articles have focused on the limitations in the management of tennis elbow using PRP injections. In addition to platelets, PRP contains various numbers of leukocytes such as monocytes and neutrophils that may influence the healing process favorably or negatively. The inclusion or exclusion of WBCs in PRP applications is a contentious issue. Inflammatory cytokines (IL-1, TNF) have been linked to leukocytes, which could have negative consequences for tissue regeneration [12,25]. Platelet activation is another hot topic in research, as the presence or absence of activation agents is expected to affect PRP's efficacy. Although activation has been proposed as a necessary step in the synthesis of PRP more than half of the studies in this analysis (57%) didn’t use the activation method. PRP stimulated by calcium chloride (CaCl2), thrombin, or CaCl2/thrombin combinations exhibited considerably higher growth factor release than non-activated PRP and platelet-poor plasma, according to a recent study evaluating the different types of PRP activating agents [26,27]. Furthermore, throughout time, different activating agents release varied concentrations of growth factors. In the first hour, the CaCl2/thrombin combination produced more growth factor release than CaCl2 alone or Type III collagen alone, but over a 24-hour period, CaCl2 activation alone provided the most growth factor releases. As a result, the activation component utilized and the interval between activation and administration have an impact on the total PRP formulation. More research is needed to standardize the use of activating chemicals in order to find the most effective activation strategy if one is required at all [28].

The limitations of this study, it was done in a single hospital, the results needed further evidence from other studies done in various clinical settings. Similarly, this is a hospital-based study, which invites a selection bias by the treating doctor. Thus, the results may not be applicable to the general population.

## Conclusions

In tennis elbow patients, PRP injections show an effective reduction in pain according to VAS and MAYO score and especially, the young age group has shown more benefit in terms of pain reduction for PRP treatment. Thus, PRP injections can be used as an important alternative to other standard regimens in and those who don't want surgery.
